# Low-level HIV-1 replication and the dynamics of the resting CD4^+ ^T cell reservoir for HIV-1 in the setting of HAART

**DOI:** 10.1186/1471-2334-8-2

**Published:** 2008-01-02

**Authors:** Ahmad R Sedaghat, Robert F Siliciano, Claus O Wilke

**Affiliations:** 1 Department of Medicine, Johns Hopkins University School of Medicine, Baltimore Maryland 21205, USA; 2 Howard Hughes Medical Institute, Baltimore Maryland 21205, USA; 3 Section of Integrative Biology, Center for Computational Biology and Bioinformatics, and Institute for Cell and Molecular Biology, University of Texas at Austin, Austin, TX 78713, USA

## Abstract

**Background:**

In the setting of highly active antiretroviral therapy (HAART), plasma levels of human immunodeficiency type-1 (HIV-1) rapidly decay to below the limit of detection of standard clinical assays. However, reactivation of remaining latently infected memory CD4^+ ^T cells is a source of continued virus production, forcing patients to remain on HAART despite clinically undetectable viral loads. Unfortunately, the latent reservoir decays slowly, with a half-life of up to 44 months, making it the major known obstacle to the eradication of HIV-1 infection. However, the mechanism underlying the long half-life of the latent reservoir is unknown. The most likely potential mechanisms are low-level viral replication and the intrinsic stability of latently infected cells.

**Methods:**

Here we use a mathematical model of T cell dynamics in the setting of HIV-1 infection to probe the decay characteristics of the latent reservoir upon initiation of HAART. We compare the behavior of this model to patient derived data in order to gain insight into the role of low-level viral replication in the setting of HAART.

**Results:**

By comparing the behavior of our model to patient derived data, we find that the viral dynamics observed in patients on HAART could be consistent with low-level viral replication but that this replication would not significantly affect the decay rate of the latent reservoir. Rather than low-level replication, the intrinsic stability of latently infected cells and the rate at which they are reactivated primarily determine the observed reservoir decay rate according to the predictions of our model.

**Conclusion:**

The intrinsic stability of the latent reservoir has important implications for efforts to eradicate HIV-1 infection and suggests that intensified HAART would not accelerate the decay of the latent reservoir.

## Background

The latent reservoir for HIV-1 in resting CD4^+ ^T cells is generated when productively infected CD4^+ ^T lymphoblasts revert back to the resting state, becoming memory T cells, instead of succumbing to viral cytopathic effects or host cytolytic effector mechanisms [1-4]. The result is a state of viral latency in resting memory CD4^+ ^T cells, cells that are extremely quiescent, with little to no transcription of HIV-1 genes [5-7]. Given that memory T cells form the basis for lifelong immunity to recall antigens, it is not surprising that the average half-life of the latent reservoir in the setting of HAART can be as long as four years [8,9]. However, the basis for the remarkable stability of the latent reservoir has remained controversial.

The two most reasonable mechanisms for maintenance of the latent reservoir in the setting of HAART are 1) replenishment by low-level viral replication [10-20] and 2) the intrinsic stability of latently infected cells (i.e. memory T cells) [8,9,21-23]. While some studies have suggested that low-level viral replication confers stability by continuously reseeding the latent reservoir despite HAART [10,19,20], other studies have provided experimental evidence at odds with a major role for viral replication in maintaining the latent reservoir [24,25]. These studies have shown that in many patients responding well to HAART, there is no evolution of drug resistance, suggesting a lack of viral replication [26]. We have previously shown that the maximal rate at which new cells enter the reservoir in the setting of HAART is extremely low [27]. These studies provide indirect evidence that intrinsic stability of memory T cells and not replenishment by ongoing viral replication is the major reason for the stability of the latent reservoir.

Mathematical models have proven useful for the analysis of several aspects of HIV-1 infection including the dynamics of viral replication [28-31], the effects of immune responses [32-35], and the mechanism of CD4 depletion [32,36-38]. We present here a mathematical analysis of CD4^+ ^T cell dynamics in the setting of HIV-1 infection in order to explore the dynamics of the latent resting CD4^+ ^T cell reservoir. We extend elegant models of HIV-1 and CD4^+ ^T cell dynamics previously described by Alan Perelson and Martin Nowak [28,32] to explore how low-level viral replication influences the observed decay of the latent reservoir in patients on HAART. A recent study [39] analyzed the persistence of the latent reservoir in the setting of HAART with a model similar to ours. However, this study [39] did not focus on the decay properties of latently infected cells in relation to low-level viral replication. Also, because the authors did not constrain the maximum amount of viral replication compatible with available experimental data from patients on HAART, this study [39] was unable to answer the clinically significant question of whether realistic levels of residual viral replication in the setting HAART affect the experimentally observed decay rate of the latent reservoir. In this study, we calculate the well-known replication threshold below which infection cannot be sustained [40] for our model, and discuss latent reservoir replenishment above and below this threshold. Having explicitly illustrated the primary factors involved in establishing and maintaining the latent reservoir, we offer the first explicit analysis of the relationship between low-level viral replication and the decay rate of the latent reservoir. Our results indicate that the impact of viral replication on the decay rate of the latent reservoir rapidly diminishes with increasing inhibition by HAART. For levels of viral replication likely to occur in the setting of HAART, our model predicts that the decay of the latent reservoir is determined by intrinsic (e.g. death rate) as well as extrinsic properties (e.g. activation rate) of latently infected cells. We further apply our theoretical analysis to patient-derived data and show that any flow of new cells into the latent reservoir by viral replication is unlikely to impact the observed, *in vivo *decay rate. Thus theoretical predictions and experimental data both suggest that the long half-life of the latent reservoir is likely attributable to the intrinsic stability and reactivation rate of latently infected T cells. As such, any possible shortening of the half-life of latently infected CD4^+^ T cells would depend on increasing the death rate or reactivation rate of these cells. These results have important implications for eradication of HIV-1 infection by conventional HAART and suggest the necessity of developing strategies to target the latent reservoir specifically by approaches either apart from or in addition to HAART intensification.

## Methods

### Mathematical model of T cell dynamics

**Figure 1:**
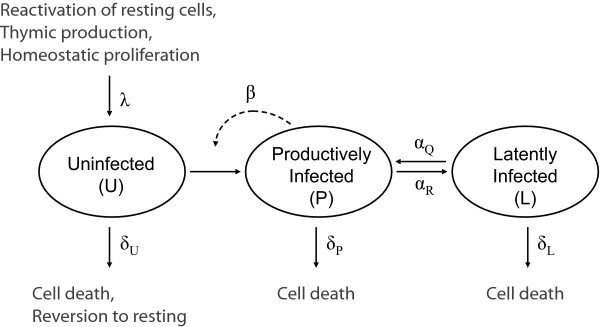
General schematic of model reflecting uninfected cells (*U*), productively infected cells (*P*) and latently infected cells (*L*). *λ *represents target T cell production. *β *reflects virus infectivity. *α*_*R *_is the rate of reversion for productively infected cells to latency. *α*_*Q *_is the activation rate of latently infected cells. Uninfected, productively infected and latently infected cells each have intrinsic death rates represented by *δ*_*U*_, *δ*_*P *_and *δ*_*L*_, respectively.

To explore the possibility that low-level viral replication reseeds the latent reservoir and thereby slows the decay of the reservoir in the setting of HAART, we used a slightly modified version of previously described mathematical models of T cell dynamics in HIV-1 infection [28,32]. By virtue of its foundation in previously described models [28,32], our model makes the same assumptions as these models: constant model parameters (e.g. viral infectivity) as well as a well-stirred, homogeneous virus and cell populations. Our modifications to these models make relatively few additional assumptions about the physiology of CD4^+ ^T cell dynamics, as described below. Our model (Figure 1) considers uninfected (activated) target cells (*U*), productively infected (activated) cells (*P*) and latently infected cells (*L*). We consider infected cells as those that contain a stably integrated HIV-1 genome. Uninfected cells are introduced into the system according to a zero order rate constant, *λ*, which represents production of target cells (e.g. by activation of resting CD4^+ ^T cells as well as thymic or homeostatic production). Uninfected cells are infected by virions to become productively infected cells at a rate proportional to a constant, *β*, which represents the virus infectivity and is a reflection of viral replication. Because free virions have a very high turnover rate and are made by productively infected cells (infected activated CD4^+ ^T cells), their dynamics closely resemble those of productively infected cells. Therefore free virions are not explicitly represented but rather are represented by productively infected cells. Thus the rate of infection is represented as a non-linear term (*βUP*) proportional to both the target (uninfected) cells and the productively infected cells [32]. Only productively infected cells replenish the latent reservoir [41]. We model the reversion of productively infected cells to latency at a rate proportional to *α*_*R*_, while latently infected cells may reactivate at a rate proportional to *α*_*Q*_.

Productively infected and latently infected cells each have intrinsic death rates represented by *d*_*P*_, and *d*_*L*_, respectively. Uninfected activated CD4+ T cells can be lost by conversion into resting T cells or by cell death. However, since resting T cells cannot be productively infected by HIV, they are irrelevant for our model, and thus we subsume any kind of loss of activated T cells under the rate constant *d*_*U*_. The model is then defined by a system of three ordinary differential equations:

$$\frac{dU}{dt}=\lambda -\delta_{U}U-\beta UP,$$

$$\frac{dP}{dt}=\beta UP-(\delta_{P}+\alpha_{R})P+\alpha_{Q}L,$$

$$\frac{dL}{dt}=\alpha_{R}P-(\delta_{L}+\alpha_{Q})L.$$

We do not incorporate other permissive cell types (e.g. macrophages) since they comprise a small fraction of all infected cells [31] and would not significantly change the overall qualitative as well as quantitative findings.

Parameter values were chosen based on previously published reports, where possible. Most productively infected cells have been shown to have extremely short half-lives [30,31], while latently infected cells have remarkable stability [8,9]. These characteristics are reflected in the death rates of productively infected cells, *δ*_*P*_, and latently infected cells, *δ*_*L*_. The rate at which target cells (uninfected activated CD4^+ ^T cells) turn over, *δ*_*U*_, represents a balance between death, proliferation and reversion to resting. In the case of no infection (i.e. in an HIV^- ^individual), the steady-state number of target cells, $\bar{U}$ is *λ*/*δ*_*U *_or *λ *= *δ*_*U*_*U*. Therefore *δ*_*U *_also represents fraction of target cells that are replaced daily. Previously published studies estimate that at most 2% of target cells are replaced daily in an uninfected individual, therefore we set *δ*_*U *_= 0.02 day^-1 ^(based on direct measurements in [42,43]). Furthermore, we find that for *δ*_*U *_= 0.02 day^-1^, our model reproduces the clinically observed, smooth rebound [29,44,45] of the viral load (reflected here by the number of productively infected cells) after interruption of HAART with little to no oscillation (data not shown). Because activated cells both proliferate rapidly and die rapidly, it is possible that the net death rate (death rate less proliferation rate) could be very small and on the order of 0.001 day^-1^. However, we do not expect that *δ*_*U *_could be much larger than our chosen value of 0.02 day^-1^. A larger value for *δ*_*U *_would be contradictory to our expectation that *δ*_*U *_<<*δ*_*P*_, which follows from the highly cytopathic nature of HIV. Regardless, while we choose *δ*_*U *_to best fit experimentally observed data, we nonetheless test values of *δ*_*U *_as low as 0.001 day^-1 ^and as large as 1.0 day^-1 ^and find our results to be robust through this entire range.

Infection of an activated CD4^+ ^T cell by HIV leads to significant dysregulation of cellular processes as the virus subverts the host cellular machinery. This dysfunction may also lead to decreased conversion to the resting state, which is reflected by the substantially lower (by several orders of magnitude) number of latently infected cells compared to productively infected cells [3]. We therefore chose a rate for conversion of productively infected cells to latency, *α*_*R*_, to reflect this experimentally observed balance. For the numerical calculations and simulations performed in this report, we used the following parameter choices (unless specified otherwise): *δ*_*P *_= 0.5 day^-1^, *δ*_*L *_= 0.0001 day^-1^, *α*_*Q *_= 0.0005 day^-1^, *α*_*R *_= *α*_*Q*_/100, and *δ*_*U *_= 0.02 day^-1 ^with *λ *constrained to 2 × 10^9 ^cells/day by the choice of *δ*_*U *_and the steady-state value of uninfected cells in the setting of no infection, which is approximately 10^11 ^cells (Table 1). However, we tested a wide range of parameter values in order to check the validity of our results (data not shown).

**Table 1:** Parameter values used for simultations and calculations

**Parameter**	**Definition**	**Values^a^**	**References**
*δ*_*P*_	Net death rate of productively infected cells	0.50 (0.5–0.693) day^-1^	[32,65,66]
*δ*_*L*_^b^	Net death rate of latently infected cells	0.0001 (0.001–0.0001) day^-1^	[32]
*δ*_*U*_	Net death rate of target cells	0.02 (1.0–0.001) day^-1^	[32,67]
*α*_*Q*_	Activation rate of latently infected cells	0.0005 (0.001–0.0001) day^-1^	[36]
*α*_*R*_^c^	Rate of reversion of productively infected cells to latency	αQ100	[3,32,68,69]
*λ*	Production rate of target cells	2 × 10^9 ^cells/day	Approximated from the steady-state condition that $\bar{U}$ = 1 × 10^11 ^in the case of no infection. Also consistent with [70]
LU¯	Ratio of latently infected cells to target cells	10^-4 ^(10^-4^–10^-6^)	[2,4]

^a^Ranges tested in parentheses

^b^Estimated based on resting cell death rate

^c^Estimated based on the ratio of latently infected cells to productively infected cells

All simulations and calculations were performed with MATLAB version 7.2.0.232. **Patient Data. **Patients' viral load records were obtained with informed consent.

## Results

### A threshold of viral replication is necessary for existence of the latent reservoir

Analysis of our model (equations 1–3 in *Methods*) leads to several predictions about the nature of HIV-1 infection and its impact on CD4^+ ^T cell dynamics. The model has two steady-state solutions: the trivial steady-state reflects conditions under which infection does not successfully occur and the system remains at the uninfected steady-state $\left(\bar{U}=\frac{\lambda }{\delta_{U}},\bar{P}=\bar{L}=0\right)$. The non-trivial steady-state values of all three state variables $\left(\bar{U},\bar{P},\bar{L}\right)$ are given by the following equations:

$$\bar{U}=\frac{A}{B\beta },$$

$$\bar{P}=\frac{\bar{L}B}{\alpha_{R}},$$

$$\bar{L}=\frac{\alpha_{R}(\lambda B\beta -\delta_{U}A)}{\beta (\delta_{P}B^{2}+\delta_{L}\alpha_{R}B)},$$

where *A *= *δ*_*L*_*δ*_*P *_+ *δ*_*P*_*α*_*Q *_+ *δ*_*L*_*α*_*R *_and *B *= *δ*_*L *_+ *α*_*Q*_. These solutions indicate that the steady-state number of latently infected cells depends on *β*, the infectivity, as does the steady state number of productively infected cells. Inspection of equation 6 immediately reveals that the non-trivial steady-state solutions (equations 4–6) are not biological (*P*, *L *≤ 0) for *β *≤ *β*_*crit*_, where

$$\beta_{crit}=\frac{\delta_{U}A}{\lambda B}.$$

Because *δ*_*P *_>> *δ*_*L*_, *α*_*Q *_>> *α*_*R*_, we can approximate *A *≈ *δ*_*P*_(*δ*_*L *_+ *α*_*Q*_) = *δ*_*P*_*B *and therefore

$$\beta_{crit}\approx \frac{\delta_{U}\delta_{P}}{\lambda }$$

A successful infection is not established (the trivial steady-state occurs) for *β *≤ *β*_*crit*_. A critical threshold dependence of the steady-state for infected cells on the infectivity was previously described for models of HIV viral and T cell dynamics [40]. Our model demonstrates a similar property and so from the condition that *L *> 0, it follows that *β *> *β*_*crit *_for a successful infection. Stability analysis shows that the non-trivial steady-state is stable (and trivial steady-state is unstable) when *β *> *β*_*crit*_, and that the converse is true when *β *≤ *β*_*crit*_.

Based on equation 8, it is apparent that *β*_*crit *_is almost entirely determined by *δ*_*U*_, *δ*_*P *_and *λ*. However, the trivial steady-state solution enforces that the number of target cells in the HIV^-^individual is *λ*/*δ*_*U*_. Because the quantity *λ*/*δ*_*U *_is roughly constant across all uninfected individuals, the primary factor influencing *β*_*crit *_is *δ*_*P*_, the death rate of productively infected cells. We find a stable, physiologically non-trivial steady state for *β *> *β*_*crit *_that reflects the need for *β *to be large enough (to generate enough productively infected cells) to overcome the large death rate of productively infected cells. Likewise, the maintenance of latently infected cells requires at least a minimum of productively infected cells sufficient for regular transitions to latency despite rapid cell death. For rate constants consistent with previously published studies (Table 1), the predicted steady-state levels of both $\bar{P}$ and $\bar{L}$ change minimally when small perturbations are made to *β*_*Untreated*_. However, both $\bar{P}$ and $\bar{L}$ rapidly drop to zero as *β *approaches *β*_*crit*_. If *β *≤ *β*_*crit*_, then the level of ongoing viral replication is unable to maintain infection and the infection subsequently decays.

In order to understand how the size of the latent reservoir relates to viral replication, we now examine the analytic relationship between infectivity and the ratio $\bar{L}/\bar{U}$, as obtained by dividing equation 6 by equation 4. From $\bar{L}/\bar{U}$ we solve for the viral infectivity

$$\beta_{Untreated}=A\left[\frac{\bar{L}}{\bar{U}}(\delta_{P}B+\delta_{L}\alpha_{R})+\alpha_{R}\delta_{U}\right]/\left(\alpha_{R}\lambda B\right)$$

in an untreated patient at steady-state. Therefore viral replication cannot replenish the latent reservoir if *β *is reduced from *β*_*Untreated *_to *β*_*crit *_where

$$\frac{\beta_{crit}}{\beta_{Untreated}}=\frac{\delta_{U}\alpha_{R}}{\frac{\bar{L}}{\bar{U}}(\delta_{P}B+\delta_{L}\alpha_{R})+\alpha_{R}\delta_{U}}.$$

Thus even in the setting of ongoing viral replication and HAART, there should be no net entry of new cells into the latent reservoir if HAART has attenuated the viral replication such that *β *≤ *β*_*crit *_(where *β*_*crit *_represents the minimum amount of viral replication necessary to maintain a latent reservoir). Below, we will argue that the experimental data constrain *β *to below *β*_*crit *_in the setting of HAART.

### Low-level viral replication does not significantly affect the decay rate of the latent reservoir

By preventing new entry into the latent reservoir, HAART induces the latent reservoir to decay at a rate that depends on the degree of inhibition of viral replication. With our model, T cell dynamics in the setting of HAART can be simulated by decreasing the infectivity, *β*, from *β*_*Untreated *_to *rβ*_*Untreated*_, where residual viral replication is represented by *r *= *β*_*HAART*_/*β*_*Untreated*_, which reflects the fraction of *β*_*Untreated *_to which viral infectivity is reduced in the setting of HAART. If we assume that *U *changes slowly relative to *P *and *L *(in the setting of HAART, the number of uninfected cells changes very little) [28], we can substitute $\bar{U}$ for *U *in equations 1 and 2 – obtaining a system of 2 linear, ordinary differential equations (for *P *and *L*). This system is characterized by two eigenvalues, where

$$-\Lambda =\frac{\frac{rA}{B}-(\alpha_{R}+\delta_{P}+B)+\sqrt{{\left(\alpha_{R}+\delta_{P}+B-\frac{rA}{B}\right)}^{2}-4A(1-r)}}{2}$$

is the asymptotic (least negative) eigenvalue and reflects the decay rate of the latent reservoir. The maximum value of Λ, Λ_*r *= 0_, is the fastest decay rate of the latent reservoir and occurs when *r *= 0, reflecting a complete inhibition of viral replication. By expanding equation 11 to zeroeth order in *α*_*R *_for *r *= 0, the maximum reservoir decay rate may be approximated as Λ_*r *= 0 _≈ *B *= *δ*_*L *_+ *α*_*Q*_. The decay rate of the latent reservoir is therefore determined primarily by the activation rate of the latently infected cells (*α*_*Q*_) as well as the net death rate of latently infected cells (*δ*_*L*_), which reflects the difference between the death rate and proliferation rate of latently infected cells. Thus the faster latently infected cells die, the faster they decay (larger *δ*_*L*_). Conversely, the faster latently infected cells divide (e.g. homeostatic proliferation), the slower they decay (smaller *δ*_*L*_). In principle, however, because resting CD4^+ ^T cells both divide and die very slowly, it is likely that *α*_*Q *_> *δ*_*L*_. Therefore, the reactivation rate of latently infected cells makes the dominant contribution to Λ, the decay rate of latently infected CD4^+ ^T cells.

**Figure 2:**
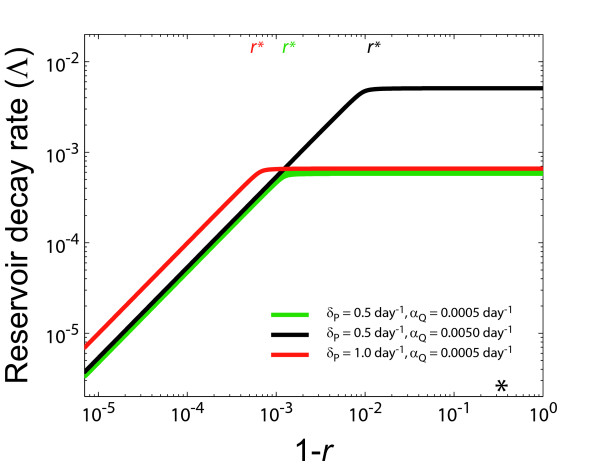
Dependence of the latent reservoir decay rate, Λ, on the degree of residual viral replication, *r*, plotted as Λ vs. 1 - *r*, for parameter values: *λ *= 2 × 10^9 ^cells/day, *α*_*R *_= *α*_*Q*_/100, *δ*_*U *_= 0.02 day^-1^, *δ*_*L *_= 0.0001 day^-1^, and $\bar{L}/\bar{U}$ = 1 × 10^-4^. We consider 3 different pairs of *δ*_*P *_and *α*_*Q *_values. The predicted value of r*, color coded for each case, is marked at the top of the plot. The marker * indicates the similar *r *= *β*_*crit*_/*β*_*untreated *_value for every pair of *δ*_*P *_and *α*_*Q*_.

Further, we observe that Λ increases as *A*(1 - *r*)/(*α*_*R *_+ *B*) until it approximately reaches its maximum (Λ_*r *= 0_) when *r *= r* ≈ (*δ*_*P *_- *δ*_*L *_- *α*_*R *_- *α*_*Q*_)/*δ*_*P*_, at which point the reservoir decay rate is virtually independent of *β *(Figure 2). This relationship between Λ and 1 - *r*, a consequence of the nonlinear structure of the model, reflects the threshold of viral replication. Once viral replication has been reduced to a level so low that the virus population cannot sustain itself anymore, the replenishment of the latent CD4^+ ^T cell reservoir through ongoing replication becomes negligible and has virtually no effect on the reservoir decay rate. This relationship between Λ and 1 - *r *also holds for other parameter combinations and the predicted reservoir decay rates closely match simulation results. (Note that for *β *> *β*_*crit*_, Λ describes the decay of the reservoir towards the new steady state, not towards zero.) In deriving an expression for the decay rate, we assume that the model parameters remain constant from when HAART is started. However, we also consider the consequences of linearizing around the post-HAART value of $\bar{U}$. At long time scales, the system might approach the post-HAART steady-state; therefore we must consider this possibility. For the case of *β *≤ *β*_*crit*_, we find that linearization around the post-HAART value of $\bar{U}$ does not significantly change our previous calculations – the reservoir continues to decay at a constant rate determined by equation 11 except that *r *= *β*_*HAART*_/*β*_*crit*_, which does not significantly change the numerical value of Λ. For the case of *β *> *β*_*crit*_, linearization around the post-HAART value of $\bar{U}$ leads to Λ ≈ 0 (because *r *→ 1 in equation 11), suggesting a very slow (almost infinitely slow) decay of the latent reservoir near the post-HAART steady-state. These calculations predict that for the case of *β *> *β*_*crit*_, the reservoir decay rate (as defined by the asymptotic eigenvalue of the equations defining our model) slows down as the post-HAART steady-state is achieved. Consistent with results that we have described in a previous study (regimes 1–3 of reference [27]), the decay of the resting CD4^+ ^T cell reservoir cannot be accurately described as an exponential decay near the post-HAART steady-state in the setting of viral replication sufficient for continual replenishment of the reservoir (*β *> *β*_*crit*_).

### Comparison with previous models of latent reservoir persistence

The decay of the latent reservoir was previously modeled by Muller *et al. *[46], who made the simplification that HAART eliminates the flow of cells into latent reservoir from the productively infected cell compartment. This simplification reduced the dynamics of the latent reservoir in the setting of HAART to a strict exponential decay dependent on only the death rate and reactivation rate of latently infected cells, and allowed Muller *et al. *to study how the reservoir decays when the cells of the latent reservoir have a distribution of reactivation rates rather than one constant reactivation rate. While quite insightful, the study by Muller *et al. *does not address the effect of viral replication on the decay of the latent reservoir by virtue of their simplifying assumption that viral replication does replenish the latent reservoir.

The persistence of low-level viremia and the latent HIV-1 reservoir in the setting of HAART was also addressed by Kim and Perelson [39], who use a simplified variation of a previous model of viral dynamics [30,31]. Kim and Perelson perform a thorough analysis of their model's parameter space to understand how various model parameters contribute to persistence of low-level viremia and a latent reservoir. Considering the effects of viral replication, Kim and Perelson find different regimes of behavior for their model (with respect to latently infected cells and viral load) that depend upon the degree of HAART efficacy (*ε*_*HAART*_) relative to a critical drug efficacy (*ε*_*crit*_) (where Kim and Perelson's efficacy, *ε*, corresponds to 1 - *r *in our model so that a larger efficacy indicates greater inhibition of viral replication). Kim and Perelson find that only when *ε*_*HAART *_> *ε*_*crit *_do both the latent reservoir and viral load decay towards zero. This finding is robust for all physiologic ranges of their model parameter values.

While offering insight into the persistence of low-level viremia and the latent reservoir, the analysis presented in Kim and Perelson does not directly address two key clinical issues: (1) whether *ε*_*HAART *_> *ε*_*crit *_in the typical HAART-treated patient and (2) how the decay rate of the latent reservoir is affected by further increasing the efficacy of HAART (e.g. by intensification of the HAART regimen) when *ε*_*HAART *_is already greater than *ε*_*crit*_. Because it has been previously suggested that intensification of HAART can increase the decay rate of the latent reservoir [20], we repeated Kim and Perelson's analysis in order to determine whether increasing *ε*_*HAART *_despite the fact that *ε*_*HAART *_> *ε*_*crit *_would predict an increase in the decay rate of the latent reservoir [20]. We find that the decay dynamics of the latent reservoir remain approximately the same regardless of *ε*_*HAART*_, as long as *ε*_*HAART *_> *ε*_*crit*_. This finding is consistent with their steady-state analysis, which found that the eigenvalue describing the long-term decay characteristics of the latent reservoir is independent of *ε *(i.e. it is unaffected by ongoing viral replication). This eigenvalue, solved for by Kim and Perelson, is consistent with the simplification of reservoir dynamics made by Muller *et al. *[46] as well as equal to our derived approximation of -Λ_*r *= 0 _(≈ -*B *= -(*δ*_*L *_+ *α*_*Q*_)) and is therefore dependent on only the net death and reactivation rate of latently infected cells. Kim and Perelson's larger model (defined by equations 1–7 of [39]) also demonstrates a threshold effect for the decay of the latent reservoir, whereby increasing HAART efficacy beyond a certain point does not accelerate the decay rate of the latent reservoir. Whether the average HAART-treated patient has reached this point of viral inhibition remains to be addressed.

### Maximal levels of viral replication consistent with patient data do not affect the decay rate of the latent reservoir

**Figure 3:**
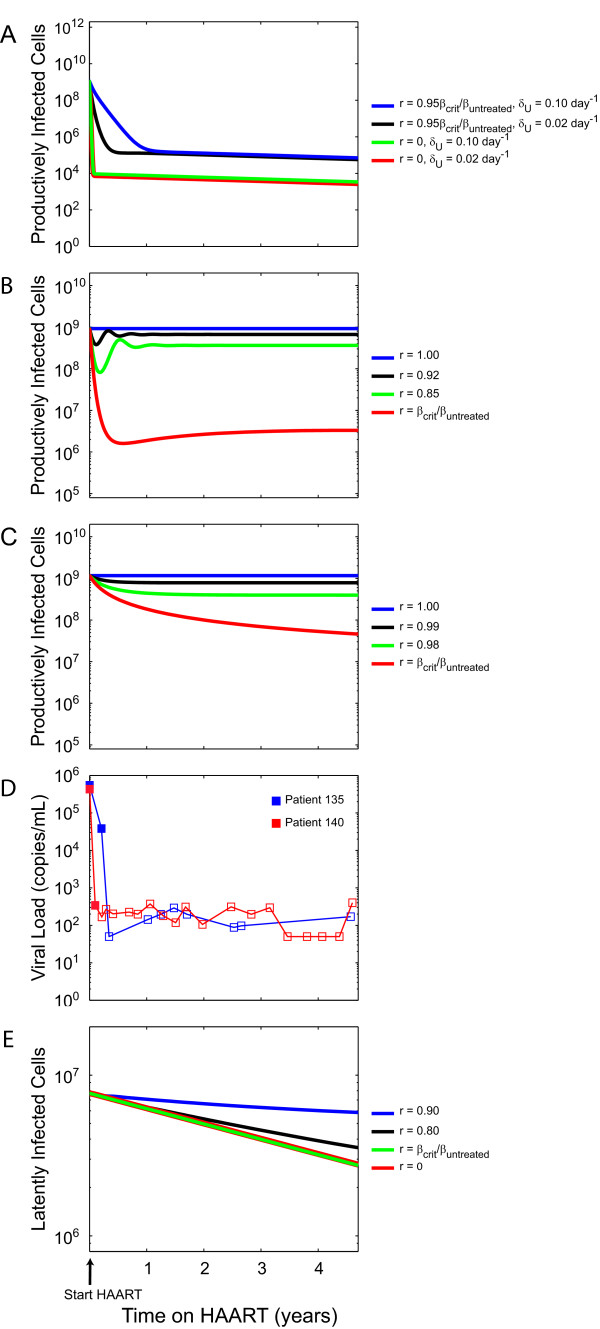
Decay of (A) productively infected cells (total number of cells) for different degrees of viral infectivity under optimal suppression (*β*_*HAART *_<*β*_*crit*_) of replication by HAART (*r *= 0 and 0.95 *β*_*crit*_/*β*_*untreated*_) for different values of *δ*_*U *_(= 0.02 day^-1 ^and 0.10 day^-1^). Decay of productively infected cells for different degrees of viral infectivity under sub-optimal suppression (*β*_*HAART *_> *β*_*crit*_) by HAART for (B) *δ*_*U *_= 0.02 day^-1 ^where *β*_*crit*_/*β*_*untreated *_≈ 0.769 and (C) *δ*_*U *_= 0.20 day^-1 ^where *β*_*crit*_/*β*_*untreated *_≈ 0.970 (D) Clinically observed decay of viral load after initiation of HAART for 2 HIV^+ ^patients with no history of drug resistance: pt. 135 (blue) and pt. 140 (red). Pt. 135's viral load has remained undetectable for an additional 5 years with only 1 blip and pt. 140's viral load has remained undetectable for 1 additional year (data not shown). Open faced markers represent undetectable viral load measurements at the limit of detection for the assay used. (E) Decay of latently infected cells (total number of cells) for different degrees of viral infectivity, where *β*_*crit*_/*β*_*untreated *_≈ 0.769. All simulations were performed with model parameter values listed in Table 1 unless otherwise specified.

In the previous sections we have shown that a threshold of viral replication is necessary for existence of the latent reservoir and that the decay rate of the reservoir is rapidly maximized with decreasing replication. To constrain viral replication in patients on HAART, we compare the behavior of the model with data from patients on HAART. If HAART reduces *β *(equal to *β*_*untreated*_) by a factor *r *= *β*_*HAART*_/*β*_*untreated *_<*β*_*crit*_/*β*_*untreated*_, our model predicts that the number of productively infected cells (and by extension, viral load) decays to zero. We find that when r is sufficiently less than *β*_*crit*_/*β*_*untreated *_(in our system *r *≲ 0.95 *β*_*crit*_/*β*_*untreated*_), the behavior of the decay of productively infected cells can be described as undergoing a rapid and approximately exponential decay, reflecting the elimination of productively infected cells produced by new infections. This initial rapid decay is followed by a slower, also approximately exponential decay, reflecting reactivation of the decaying latently infected cell pool (Figure 3A), towards zero. This result is consistent with clinically observed decay of viral load in HIV^+ ^patients as they start HAART with the exception that our model does not predict a second phase decay. The latter is absent because we include only CD4^+ ^T cell compartments in our model. This decay behavior of the productively infected cell pool demonstrated by our model occurs regardless of the parameter choices tested, as long as *r *<*β*_*crit*_/*β*_*untreated*_. If HAART reduces *β *such that *r *≳ *β*_*crit*_/*β*_*untreated *_(indicating a suboptimal HAART regimen that cannot fully suppress the infection), then we must consider two cases. In our model, we find two regimes of behavior for the decay of productively infected cells that depend on the value of *δ*_*U *_(the rate at which target cells are removed from the system).

The dependence of the system's qualitative behavior on *δ*_*U *_is robust to choice of other model parameters. With the other model parameter values that we use, if *δ*_*U *_≲ 0.1035 day^-1 ^then suboptimal HAART causes the productively infected population to rapidly decay but subsequently rebound, undergoing a dampened oscillation (see Appendix) towards a final steady-state that is greater than zero if *r *> *β*_*crit*_/*β*_*untreated *_or a zero steady-state if *r *= *β*_*crit*_/*β*_*untreated *_(Figure 3B). In contrast, if *δ*_*U *_≳ 0.1035 day^-1 ^then in the setting of a suboptimal HAART regime productively infected cells undergo a smooth but slow sub-exponential decay (see Appendix) toward a greater-than-zero final steady state if *r *> *β*_*crit*_/*β*_*untreated *_or a zero steady-state if *r *= *β*_*crit*_/*β*_*untreated *_(Figure 3C).

Previous longitudinal studies of viral load in HIV^+ ^patients starting HAART have observed a characteristic decay of the viral load [4,18,30,31] consistent with the patients we have observed (Figure 3D). In these patients, who are typical of patients responding well to HAART, the viral load is observed to make a rapid, approximately exponential decay, which reflects decay of productively infected cells. In these patients, who are typical of patients responding well to HAART, the viral load is observed to make a rapid, approximately exponential decay, which reflects decay of productively infected cells. The viral load decays to below the limit of detection, reaching a quasi-steady-state that has recently been observed experimentally [47] and is consistent with a much slower phase of decay (half-life of >67 weeks). These observed dynamics of low-level viremia most likely represents reactivation from the decay of long-lived reservoirs for HIV-1 (e.g. resting CD4^+ ^T cells). Clinically, the viral load of a patient who responds well to HAART (i.e. a successful and optimal HAART regimen) never makes a sustained rebound after the initial, rapid drop. In our simulations (Figure 3A), we observe this characteristic decay of productively infected cells only for values of *β *up to *β *≈ *β*_*crit*_. Our model therefore conservatively constrains *β*_*HAART *_≲ *β*_*crit *_based on the available experimental data, but in fact *β*_*HAART *_may be as low as zero.

For almost all values of *r *that we tested, the decay of latently infected cells, *L*, towards a new steady-state (that depends on the value of *r*) or eradication was the same (Figure 3E). For the case where *r *> *β*_*crit*_/*β*_*untreated*_, the decay of the reservoir can be described as approximately exponential only initially. However, at some point when the reservoir size approaches the non-zero post-HAART steady-state, the reservoir decay becomes sub-exponential and can no longer be described with the asymptotic eigenvalue. This is apparent by the curves in Figure 3E for *r *> *β*_*crit*_/*β*_*untreated*_, which initially decay along the exponential decay curves where *r *≤ *β*_*crit*_/*β*_*untreated *_but subsequently separate as sub-exponential decays towards the non-zero post-HAART steady-state.

Based on the constraint that $r=\frac{\beta_{HAART}}{\beta_{Untreated}}≲\frac{\beta_{crit}}{\beta_{Untreated}}$ predicted by our model, *r *may range between 0.1428 to 0.9940 for values of *δ*_*U *_ranging from 0.001 day^-1 ^to 1.0 day^-1^. For these values of *r*, we calculate that the decay of the latent reservoir observed in patients who are successfully treated with HAART is essentially at its maximum value Λ_*r *= 0 _(which occurs when there is no viral replication) and is therefore independent of any ongoing, low-level viral replication.

For these calculations, we used parameter values consistent with previously reported values (Table 1) and a 44 month half-life for the latent reservoir [8,9]. Previous studies have estimated the fraction of resting CD4^+ ^T cells that consists of latently infected cells to be on the order of 1 in 10^4 ^[2-4]. Although *U *represents target cells (i.e. activated CD4^+ ^T cells) in our model, we estimate $\bar{L}/\bar{U}$ = 10^-4 ^because the number of activated CD4^+ ^T cells (e.g. CD69^+^, CD25^+ ^and/or HLA-DR^+^) is on the same order of magnitude as the number of resting CD4^+ ^T cells in patients on HAART [48,49]. Alternative parameter choices also consistent with previously reported values, but for which the latent reservoir decays with a half-life not equal to 44 months (e.g. 6 months, 12 months) or alternative values of $\bar{L}/\bar{U}$ (e.g. 10^-5^–10^-6^) show that Λ is nonetheless very close to Λ_*r *= 0_.

### Implications for eradication of the latent reservoir

**Figure 4:**
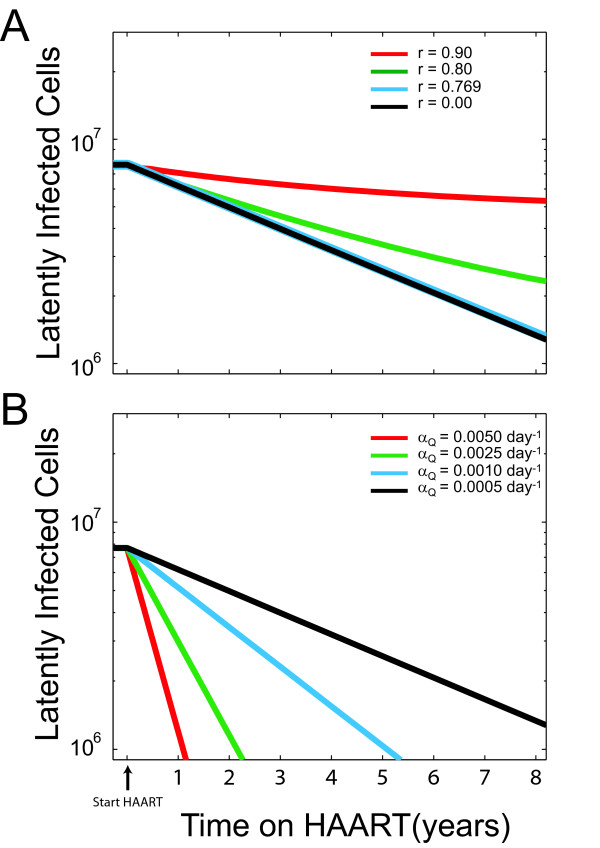
Decay of the latent reservoir as a function of time for (A) different degrees of HAART suppression of viral infectivity, *r *= 0 (black), 0.769 (*β*_*crit*_/*β*_*untreated*_) (blue), 0.80 (green), 0.90 (red); and (B) different activation rates, *α*_*Q *_= 0.0005 day^-1 ^(black), 0.001 day^-1 ^(blue), 0.0025 day^-1 ^(green) and 0.005 day^-1 ^(red). Parameter values, unless otherwise specified, are: *λ *= 2 × 10^9 ^cells/day, *β *= 0, *α*_*Q *_= 0.0005 day^-1^, *α*_*R *_= *α*_*Q*_/100, *δ*_*U *_= 0.02 day^-1^, *δ*_*P *_= 0.50 day^-1^, *δ*_*L *_= 0.0001 day^-1 ^and $\bar{L}/\bar{U}$ = 1 × 10^-4^.

For values of *β *that are consistent with clinically observed viral load decay characteristics in the setting of HAART, the reservoir decay rate remains essentially constant at Λ_*r *= 0 _Because Λ_*r *= 0 _≈ *B *= *α*_*Q *_+ *δ*_*L*_, the major determinants of the reservoir decay rate are the activation rate and the intrinsic death rate of latently infected cells. We used our model to compare the potential for increasing the decay rate of the latent reservoir by enhanced inhibition of viral infectivity, which would occur in the setting of intensified HAART, to enhanced reactivation of latently infected cells, a therapeutic option that is currently being developed [50-52]. Figure 4A describes the case of viral inhibition by HAART. For non-suppressive reductions in the infectivity (*r *> *β*_*crit*_/*β*), the decay dynamics of the latent reservoir diverge from an exponential decay and asymptotically move toward a non-zero steady-state number of latently infected cells. However, for a suppressive reduction in infectivity (*r *<*β*_*crit*_/*β*), the latent reservoir decays exponentially with a rate constant equal to *B *= *α*_*Q *_+ *δ*_*L *_towards zero latently infected cells (Figure 4A). Our model therefore predicts that any drug or drug combination reducing HIV infectivity by a factor *r *<*β*_*crit*_/*β *will cause the latent reservoir to decay with the same dynamics. We do not know the value of *r *for current HAART regimens, although our analysis suggests that *r *<*β*_*crit*_/*β*. Nonetheless, we must consider the possibility that *r *> 0, in which case intensified HAART may further reduce *r *without hastening the decay of the latent reservoir. It is also possible that standard HAART regimens reduce infectivity such that *r *= 0 (i.e. *β*_*HAART *_= 0), in which case intensification of standard HAART regimens would have no conceivable benefit.

Unlike the case of decreasing infectivity, we find that increasing reactivation of latently infected cells will greatly increase the decay of the reservoir (Figure 4B). This result suggests that while the full potential of HAART on the reservoir decay rate has been achieved, enhanced reactivation of latently infected cell may offer another approach to accelerating the decay rate of the latent reservoir.

## Discussion

Despite rapid decay of free virus and of most productively infected cells after the initiation of HAART, the latent reservoir for HIV-1 in resting CD4^+ ^T cells remains as an inducible source of viremia that can potentially lead to rebound in plasma virus levels upon discontinuation of HAART. The latent reservoir is extremely stable, with a half-life of 44 months [8,9,53]. Because the latent reservoir represents a major barrier to eradication, many studies have focused on developing methods to purge the reservoir [50-52]. Successful eradication of the latent reservoir, however, depends on understanding the mechanism underlying its remarkable stability.

The two factors believed to be most important in determining the decay rate of the latent reservoir are 1) the degree of replenishment of the reservoir by ongoing viral replication that continues despite HAART and 2) the intrinsic stability of resting memory T cells, the cells that harbor latent HIV-1. How each factor contributes to stabilizing the reservoir has important implications in development of methods to eradicate the reservoir. Evidence for replenishment of the reservoir in the setting of HAART comes from clinical studies showing accelerated reservoir decay for patients on an intensified HAART regimen over that observed in patients on standard HAART regimen [10,19,20]. The results of these studies suggest that standard HAART does not stop all replication, raising the possibility that ongoing replication could play a role in maintaining the latent reservoir. However, there are several lines of evidence that argue against ongoing viral replication in patients responding well to HAART. First, a meta-analysis of a large number of clinical trials of antiretroviral drugs has shown that the most successful HAART regimens are those that include two drugs with an extremely low genetic barrier to resistance (efavirenz and lamivudine or emtricitabine) (John A. Bartlett, *12*^*th *^*Conference on Retroviruses and Opportunistic Infections*, 2005). Second, patients who demonstrate consistent suppression of viremia on HAART do not develop drug-resistant virus [9,25,26,54]. Third, direct examination of the residual viremia has shown a static evolutionary pattern, with no evidence of evolution even during blips [24,26]. Fourth, in many patients on HAART, most of the residual viremia is comprised of a small number of viral clones that are released into the plasma for long periods of time without evolution, suggesting a mechanism of persistence other than viral replication [55]. Using patients who demonstrate these predominant plasma clones, we have quantitatively constrained the rate at which new cells are introduced in the latent reservoir [27]. This study confirmed the minimal contribution of new entrants into the latent reservoirs of these patients. The lack of evidence for replication-driven replenishment of the latent reservoir suggests that intrinsic stability of the reservoir is the most significant factor in determining the observed reservoir decay rate [8,9,21-23].

There is no direct evidence, however, against the ability of low-level replication in the setting of HAART to stabilize the latent reservoir. In this study, we extend a previously described mathematical model of HIV dynamics by incorporating latently infected cells in order to understand the effect of low-level viral replication on the decay of the latent reservoir. Previously, more complicated models of T cell dynamics in the setting of HIV infection have been published [33,56]. However, these models often incorporate physiologic processes that have not yet been well characterized in the experimental literature. Because the kinetics of these processes have not been quantified experimentally, it is difficult to infer much information from these models. By contrast, we use a more concise model of T cell dynamics that incorporates widely accepted physiologic relationships between different T cell populations. While all aspects of T cell dynamics are not yet known, the kinetics of the T cell activation processes included in our model represent the best characterized in the experimental literature. Based on the previous track record of similar models and the preponderance of data supporting the structure as well as results of our model, we believe that our model provides insight into the effects of low-level viral replication in the setting of HAART.

Like previous models, in our model a threshold of viral replication must be surpassed to overcome the cytopathic effects of HIV-1 infection [40]. Our model predicts two regimes for how ongoing viral replication impacts the decay rate of the latent reservoir. In the first regime, high levels of ongoing viral replication slow the decay rate of the latent reservoir; in this regime, attenuation of viral replication would hasten the decay of the latent reservoir. In the second regime, lower levels of viral replication do not significantly affect the decay rate of the latent reservoir. The low frequency at which a productively infected cell transitions to latency requires that many productively infected cells be present before one latently infected cell is produced. In this regime, the reduced number of productively infected cells is insufficient to consistently replenish the latent reservoir and therefore further attenuation of viral replication would not increase the decay rate of the latent reservoir. Based on previously reported model parameter values, we numerically explored the boundaries of these regimes by calculating the predicted reservoir decay rate for various levels of viral replication in the setting of HAART. We found that the impact of viral replication on the reservoir decay rate is rapidly minimized with decreasing viral replication. It has been hypothesized that there exist other cellular reservoirs for HIV-1, most likely in the form of monocytes and tissue macrophages [57,58], that are possibly maintained in the setting of HAART by ongoing viral replication. While these reservoirs may potentially be purged faster with intensification of HAART, our study shows that the decay rate of the resting CD4^+ ^T cell reservoir cannot be increased in this manner.

Based on our calculations of r* (the reduction in *β *necessary before the decay of the latent reservoir becomes independent of ongoing replication; Figure 2) and our model predictions for the dynamics of viral load (reflected by productively infected cells), we would intuitively expect standard HAART to reduce viral replication into the regime where residual replication would have an insignificant effect on the reservoir decay rate. To test this theory, we compared previously reported patient data to the behavior of our model for different levels of HAART efficacy. Our analysis found that the behavior of the model can quite clearly reproduce experimental findings observed in the setting of suppressive HAART. In this regime of low-level viral replication, our model predicts that viral replication does not significantly affect the decay rate of the latent reservoir.

If a small amount of viral replication is insufficient to affect the decay rate of the latent reservoir, then it may be asked why complete suppression should be the goal of HAART. Despite our analysis suggesting that low-level viral replication does not affect the decay rate of the latent reservoir, ongoing viral replication in the setting of intense selective pressure by HAART may lead to selection of drug resistant viruses, which could potentially return the infection back or close to the original steady-state achieved by wild-type virus in the absence of drugs. Thus achieving suppression of replication, while not necessarily accelerating reservoir decay, is critical to preventing the evolution of drug resistance [59]. While a lack of clinically observed drug resistance in patients on HAART has been thought to reflect a lack of viral replication, a lack of viral evolution may also be observed if a viable drug-resistant mutant requires too many mutations to occur in a reasonable amount of time. The rare and unlucky HIV^+ ^patient may archive an HIV mutant that is one mutation away from becoming completely drug resistant, which may occur from possible low-level viral replication. However, there is a highly significant correlation between patient non-adherence and development of multi-drug resistance, and patient non-adherence remains by far the most likely cause for the emergence of multi-drug resistance. Furthermore, because patient adherence is self-reported, non-adherence remains the most likely cause of multi-drug resistance even in reportedly adherent patients. Therefore, prevention of multi-drug resistance is not a validated rationale for intensified HAART [60]. However, future work will be helpful in understanding whether low-level viral replication in the setting of HAART may lead to multi-drug resistance.

## Conclusion

Our results have several implications. Standard antiretroviral medications have some toxicities and side effects, which may motivate patient non-adherence leading to the subsequent development of drug resistance. Intensification of HAART may also lead to intensification of drug toxicities and adverse effects. Therefore implementation of an intensified HAART regimen can only be justified if the benefit would outweigh the cost in patient morbidity. If there is essentially no risk of drug resistance in compliant patients on standard HAART regimens, then intensified HAART would have little to no benefit towards maintaining suppression of viremia. If the decay rate of the latent reservoir in patients on standard HAART regimens has reached a maximum, then an intensified HAART regimen would have no benefit toward eradication of the latent reservoir – suggesting that other approaches would be necessary for eradication of the reservoir. In contrast, HAART simplification (reduction in the number of drugs) has been recently discussed as an option for reducing the burden of treatment on patients who have already achieved sustained virologic suppression [61]. Based on our findings, we expect that a simplified HAART regimen would maintain the decay rate of the latent reservoir as long as long virologic rebound is not observed. Yet whether such a simplified HAART regimen would provide effective protection against resistance mutations is a different question, which is beyond the scope of our analysis. Our results suggest that the intrinsic dynamic properties of the reservoir are the primary factors that determine the decay rate of the latent reservoir. In particular, we find that the activation rate and intrinsic death rate of the latently infected cells primarily determine the decay rate of the latent reservoir and may serve as alternative targets to be exploited for accelerating the decay of the reservoir [62-64].

Eradication of the HIV infection is and will remain an extremely difficult undertaking. The complex dynamic relationship between viral replication, the latent reservoir, and the evolution of drug resistance, as well as management of these issues in the context of minimizing patient morbidity poses a daunting task for health care providers. Insight into these dynamic relationships will provide a greater understanding of the underlying principles guiding patient care and will hopefully lead to new approaches for achieving the ultimate goal of HIV eradication.

## Competing interests

The author(s) declare that they have no competing interests.

## Authors' contributions

ARS, RFS and COW conceived the model. ARS and COW designed and analyzed the model. ARS, RFS and COW interpreted the results of the model and wrote the manuscript.

## Appendix

We assume that the number of latently infected cells does not change significantly over the time scale of oscillations that occur immediately after initiation of HAART. Therefore we set *dL*/*dt *= 0 and our model defined by equations 1–3 reduces to

$$\frac{dU}{dt}=\lambda -\delta_{U}U-\beta UP$$

$$\frac{dP}{dt}=\beta UP-(\delta_{P}+\alpha_{R})P+\alpha_{Q}L.$$

We linearize equations A1 and A2 around the pre-HAART steady-state values $\bar{U}$ and $\bar{P}$ to obtain:

$$\frac{d}{dt}=\left(\begin{array}{c} U\\ P \end{array}\right)=\left(\begin{array}{cc} -\delta_{U}-\beta \bar{P} & -\beta \bar{U}\\ \beta \bar{P} & \beta \bar{U}-(\delta_{P}+\alpha_{R}) \end{array}\right)\cdot \left(\begin{array}{c} U\\ P \end{array}\right)$$

Based on the pre-HAART steady-state values $\bar{U}$ and $\bar{P}$ described in equations 4 and 5 and the approximation that *A*/*B *≈ *δ*_*P *_(described in the main text), we can simplify equation A3 to

$$\frac{d}{dt}\left(\begin{array}{c} U\\ P \end{array}\right)=\left(\begin{array}{cc} -\delta_{U}-\frac{\bar{L}}{\bar{U}}\frac{A}{\alpha_{R}} & -\delta_{P}\\ \frac{\bar{L}}{\bar{U}}\frac{A}{\alpha_{R}} & -\alpha_{R} \end{array}\right)\cdot \left(\begin{array}{c} U\\ P \end{array}\right).$$

We determined the two eigenvalues of this system, which reflect the kinetic properties of the dynamics of *U *and *P*:

$$\Lambda_{1,2}=-\frac{1}{2}\left[\delta_{U}+\frac{\bar{L}}{\bar{U}}\frac{A}{\alpha_{R}}+\alpha_{R}\pm \sqrt{{\left(\delta_{U}+\frac{\bar{L}}{\bar{U}}\frac{A}{\alpha_{R}}-\alpha_{R}\right)}^{2}-4\delta_{P}\frac{\bar{L}}{\bar{U}}\frac{A}{\alpha_{R}}}\right].$$

If *U *and *P *change without oscillations, then the eigenvalues of this system defined by equation A5 must have no imaginary parts. This restricts

$${\left(\delta_{U}+\frac{\bar{L}}{\bar{U}}\frac{A}{\alpha_{R}}-\alpha_{R}\right)}^{2}-4\delta_{P}\frac{\bar{L}}{\bar{U}}\frac{A}{\alpha_{R}}\geq 0,$$

and therefore

$$\delta_{U}\geq \alpha_{R}-\frac{\bar{L}}{\bar{U}}\frac{A}{\alpha_{R}}+2\delta_{P}\sqrt{\frac{\bar{L}}{\bar{U}}\frac{\left(\alpha_{Q}+\delta_{L}\right)}{\alpha_{R}}}.$$

Based on numerical values of our model parameters, we determine that for approximately *δ*_*U *_≤ 0.1035, there will be no oscillations in the dynamics of *U *and *P*. The dominant factor that determines the lower bound on *δ*_*U *_in equation A7 is $2\delta_{P}\sqrt{\frac{\bar{L}}{\bar{U}}\frac{\left(\alpha_{Q}+\delta_{L}\right)}{\alpha_{R}}}$, which is several orders of magnitude larger than the other components of *δ*_*U*_. Examination of this expression reveals that the lower bound on *δ*_*U *_depends approximately linearly on *δ*_*P*_. The other major factor capable of producing large variation in the lower bound on *δ*_*U *_is the quantity $\frac{\bar{L}}{\bar{U}}$, which is variable from person to person and is also viral strain dependent. Over time it is also likely that the activation rate of latently infected cells decreases. Taking the limit as *α*_*Q *_goes to zero, we find that the lower bound on *δ*_*U *_is not significantly affected – decreasing by slightly more than a factor of two, from 0.1035 to 0.0437.

## Pre-publication history

The pre-publication history for this paper can be accessed here:


